# Carbapenem Antibiotics Versus Other Antibiotics for Complicated Intra-abdominal Infections: a Systematic Review and Patient-Level Meta-analysis of Randomized Controlled Trials (PROSPERO CRD42018108854)

**DOI:** 10.1007/s11605-023-05651-7

**Published:** 2023-03-22

**Authors:** Artur Rebelo, Laura Schlicht, Jörg Kleeff, Christoph W. Michalski, Max Heckler

**Affiliations:** 1 Department of Visceral, Vascular and Endocrine Surgery, University Hospital Halle (Saale), Halle, Germany; 2 Department of General- and Visceral Surgery, University Hospital Ulm, Ulm University, Ulm, Germany; 3 Department of General-, Visceral- and Transplantation Surgery, University Hospital Heidelberg, Im Neuenheimer Feld 420, 69120 Heidelberg, Germany

**Keywords:** Infections, Abdominal, Antibiotics, Surgery

## Abstract

**Background:**

The treatment of complicated intra-abdominal infections remains a challenge. Both optimal medical and surgical therapy (i.e., source control) are needed to achieve low mortality and morbidity. The objective of this systematic review and meta-analysis is to determine the impact of carbapenem antibiotic therapy compared to other antibiotics in complicated intra-abdominal infections (secondary peritonitis) with an emphasis on mortality and postoperative complications.

**Methods:**

A systematic literature search from PubMed/Medline and Web of Science databases was carried out. The last search was conducted in August 2022. PRISMA guidelines were followed. Pre-defined outcomes were mortality, treatment success, treatment failure, and adverse events.

**Results:**

Ten randomized controlled trials, published from 1983 to 2013 with a total of 2377 patients (1255 patients in the carbapenem antibiotics group and 1122 in the control group), were identified. A meta-analysis comparing patients undergoing carbapenem antibiotic therapy and patients receiving other antibiotics was performed. No significant difference regarding mortality (OR 1.19, 95% CI [0.79; 1.82], *p* = 0.40), treatment success (OR 1.17, 95% CI [0.72; 1.91], *p* = 0.53), and treatment failure (OR 0.84, 95% CI [0.48; 1.45], *p* = 0.52) was observed. Carbapenem therapy was associated with fewer adverse events compared to therapy with other antibiotics (OR 0.79, 95% CI [0.65; 0.97], *p* = 0.022).

**Conclusion:**

There is currently no evidence that carbapenem antibiotics are superior in terms of mortality, and success or failure for the treatment of complicated intra-abdominal infections (secondary peritonitis). The rate of adverse events is lower under carbapenem therapy compared to control antibiotics.

**Trial Registration:**

PROSPERO 2018 CRD42018108854.

**Supplementary Information:**

The online version contains supplementary material available at 10.1007/s11605-023-05651-7.

## Background

Complicated intra-abdominal infections (CII) and secondary peritonitis (SP) are characterized by a loss of bowel wall integrity and subsequent abscess formation and/or spread into the peritoneal cavity. The etiology is either spontaneous due to perforation or trauma, or postoperative, usually in the context of anastomotic leakage. CII can originate from the upper and lower gastrointestinal (GI) tract and from the hepatobiliary system. The most common sources of CII include perforated appendicitis, perforated diverticulitis of the sigmoid colon, gastric and duodenal ulcers, and perforated cholecystitis.

CII are surgical emergencies associated with substantial mortality and morbidity, requiring immediate multimodal treatment: Source control strategies are dependent on the anatomical location and the etiology and include emergency laparotomy/laparoscopy, percutaneous drainage, and endoscopic interventions. The aim of these strategies is to close mucosal breaches, drain infected collections/abscesses, and remove necrotic tissue.

Early empiric systemic antibiotic (AB) therapy is the other cornerstone of the therapeutic approach.

CII are usually characterized by polymicrobial contamination of the peritoneal cavity, requiring antimicrobial agents with a broad spectrum—covering coliforms and anaerobes among others—and a good tissue penetration. In addition, patient-related factors such as age, performance status, immunocompromised state, and previous antibiotic therapy need to be taken in account before the start of treatment.^1^

Once the results of microbiological culture and susceptibility are obtained, a switch to a targeted antimicrobial therapy is possible and advised.

Early-stage empiric antibiotic (AB) treatment usually consists of broad spectrum single or multi drug regimens, without robust evidence for one combination of drugs over another.

In this context, carbapenem antibiotics such as meropenem or imipenem—classically used in critically ill patients—have been tested against other therapies, like broad spectrum beta lactam antibiotics or combinations of cephalosporin antibiotics plus metronidazole, in several studies. To date, no comprehensive meta-analysis on the effectiveness of carbapenems versus other antibiotics for CII has been conducted.

The objective of this systematic review and meta-analysis is to determine the impact of carbapenem AB therapy when compared to other antibiotics in CII, to guide future clinical decision-making.

## Methods

The literature search and data analysis were conducted in accordance with the PRISMA guidelines.^2^ The study has been registered in the PROSPERO database.^3^

## Search Strategy

The PubMed/Medline and Web of Science databases were searched for this study through its respective online search engines. The search was performed on studies published between 1983 and a defined search date. The last search was conducted on 01.08.2022. The following search strategy was used: (((peritoniti*) OR “Peritonitis”[MeSH]) OR “Intraabdominal Infections”[MeSH]) AND antibio* OR “Anti- Bacterial Agents/therapy”[MeSH] AND ((“1983/01/01”[PDat]: “2022/08/01”[PDat])). Furthermore, the reference lists of the included studies were manually searched to find relevant articles. Abstracts and full-text reviews were evaluated independently in an unblinded standardized manner by two authors (LS and MH) to assess eligibility for inclusion or exclusion. Disagreements between reviewers were resolved by consensus; if no agreement could be reached, a third reviewer (AR) decided whether to include the respective study.

## Inclusion and Exclusion Criteria

**Fig. 1:**
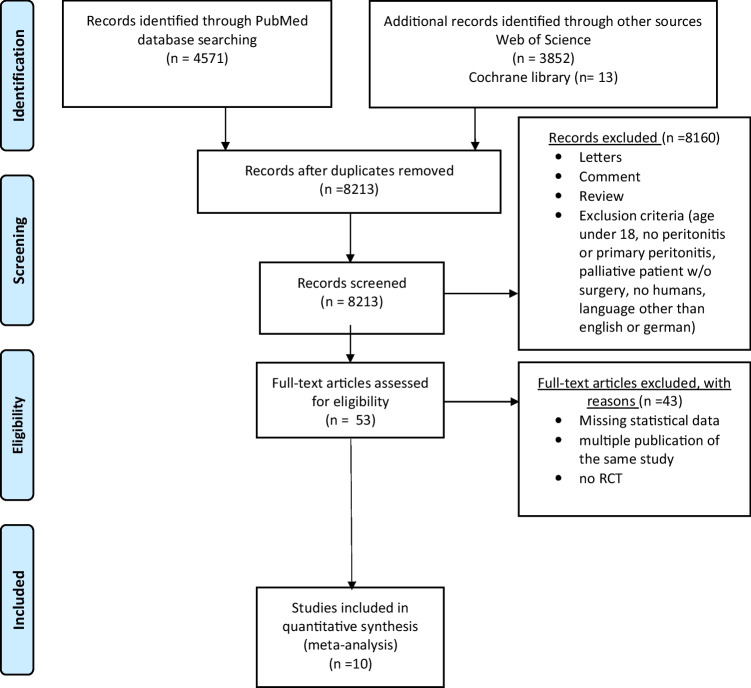
PRISMA flow diagram

Articles in English and German were considered. Randomized controlled trials reporting on patients over 18 years with secondary peritonitis that received surgery were included. Studies with patients under 18 years with no peritonitis or primary peritonitis, palliative patients, and studies with no explicit information on antibiotic therapy with carbapenem antibiotics and control group were excluded. Animal studies were excluded. Also, studies with an irrelevant abstract or title were excluded, as were reviews, case reports, case series with less than five patients, comments, and letters. Details of the study selection process are summarized in a flowchart (Fig. 1).

## Data Collection

Studies were analyzed, and data was extracted separately by two authors and presented in a tabular fashion. Individual patient level data were extracted, and odds ratios were calculated for each study and outcome. The following descriptive data was documented for each selected study: first author, year of publication, and sample size. The following predefined outcomes were also extracted: mortality, treatment success (proportion of cases that successfully completed treatment without bacteriological evidence of failure or defined as the included studies), treatment failure (proportion of cases that failed to complete treatment without bacteriological evidence of failure or defined as the included studies), and adverse events for two groups: carbapenem antibiotics (CE) and other antibiotics (OA). Risk of bias was assessed by two authors using the Cochrane Collaboration risk-of-bias tool for RCTs.

## Statistical Analysis

R version 4.1.0 (R Project for Statistical Computing, Vienna, Austria) and the meta-analysis package meta for 1.9–9 were used for statistical analysis. A random effects model was used. The magnitude of the effect estimate was visualized by forest and funnel plots. An odds ratio (log scale) (OR) was calculated for binary data. The 95% confidence interval (CI) was reported for each outcome.

## Results

Among 8213 articles, 10 RCTs fulfilling the inclusion criteria were identified and included in the meta-analysis^4–14^ (Fig. 1). Inclusion period varied from 1983 to 2013. Study characteristics are depicted in Table 1. Within the included studies, a total of 2377 patients (1255 patients in the carbapenem antibiotics group and 1122 in the control group) were identified. Duration of follow-up ranged from 2–4 to 4–6 weeks after end of treatment and was reported for 8 of the 10 RCTs (Supplemental Table 1).

**Table 1:** Study characteristics

Author	Year	Patient intervention/control	Intervention	Control	Endpoints
Gonzenbach	1984	47/46	Imipenem	Netilmicin/clindamycin	Adverse events, failure, success
Brismar	1992	58/55	Imipenem/cilastatin	Piperacillin/tazobactam	Mortality, adverse events, failure, success
Angeras	1996	258/257 (ITT), 161/145	Imipenem/cilastatin	Cefuroxim/metronidazol	Mortality, adverse events, failure, success
Kempf	1996	43/40	Meropenem	Cefotaxim/metronidazol	Mortality, adverse events, failure, success
Wilson	1997	132/134 (ITT)	Meropenem	Clindamycin/tobramycin	Mortality, adverse events, failure, success
Jaccard	1998	83/76	Imipenem-cilastatin	Piperacillin-tazobactam	Mortality, adverse events, failure, success
Solomkin	2001	270/259 (ITT)	Imipenem/cilastatin	Clinafloxacin	Mortality, adverse events, failure, success
Solomkin	2003	203/193	Ertapenem	Piperacillin/tazobactam	Mortality, adverse events, failure, success
Catena	2013	71/71	Ertapenem	Ampicillin-sulbactam	Mortality, adverse events, failure, success
Lucasti	2013	90/87	Meropenem	Metronidazol/ceftazidim/avibactam	Mortality, adverse events, failure, success

Data on the source of infection could be extracted for 8 studies with 2226 patients. Perforated/complicated appendicitis was the origin of the complicated intra-abdominal infection in 1074 patients (48%, ranging from 37 to 60%). Seventeen percent (*n* = 370) of patients had an infection originating from the colon and 8% (*n* = 182) of infections were due to perforated stomach/duodenal ulcerations. Further sources included gallbladder/biliary (7%, *n* = 161) and small bowel (4%, *n* = 91). Detailed information on the origin of infection for each study is given in Table 2.

**Table 2:** Origin of intra-abdominal infection. *s.p*., safety population

Author	Year	Total	Appendix	Appendix (%)	Colon	Colon (%)	Small bowel	Small bowel (%)	Stomach/duodenum	Stomach/duodenum(%)	Gallbladder/biliary	Galbladder/biliary (%)	Other/unspecified	Other (%)
Gonzenbach	1984	93	53	57%	15	16%	4	4%	3	3%	8	9%	10	11%
Angeras	1996	515	202	39%	93	18%	0	0%	51	10%	15	3%	154	30%
Kempf	1996	83	31	37%	27	33%	0	0%	25	30%	0	0%	0	0%
Wilson	1997	266	141	53%	14	5%	0	0%	11	4%	6	2%	94	35%
Solomkin	2001	529	249	47%	112	21%	45	9%	21	4%	41	8%	61	12%
Solomkin	2003	396	236	60%	72	18%	24	6%	17	4%	23	6%	24	6%
Catena	2013	142	66	46%	19	13%	1	1%	2	1%	54	38%	0	0%
Lucasti	2013	202 (s.p.)	96	48%	18	9%	17	8%	52	26%	14	7%	5	2%
*Sum*		*2226*	*1074*	*48%*	*370*	*17%*	*91*	*4%*	*182*	*8%*	*161*	*7%*	*348*	*16%*

**Fig. 2:**
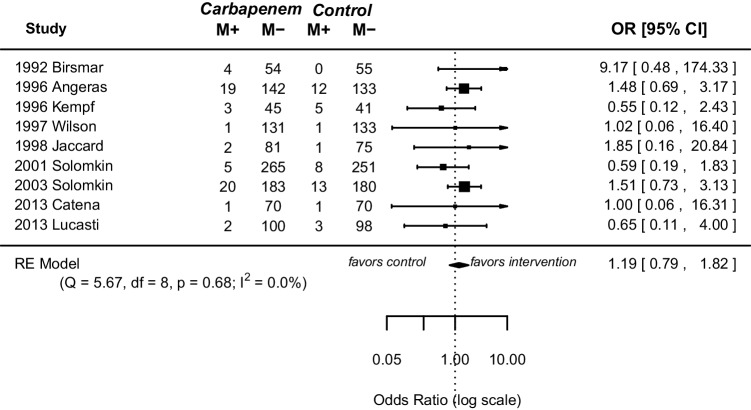
Forest plot of pooled odds ratio with 95% CI for CE vs OA regarding mortality

Nine out of 10 RCTs reported on mortality. Fifty-seven and 44 patients died in the carbapenem and control group. Meta-analysis showed no significant difference between the carbapenem group and the control group (OR 1.19, 95% CI [0.79; 1.82], *p* = 0.40) (Fig. 2). Study heterogeneity was low (*I*^2^ = 0.00, *p* = 0.68). Five studies had detailed reports on the cause of death, with most deaths associated with cardiac failure and septic shock (Supplemental Table 2).

**Fig. 3:**
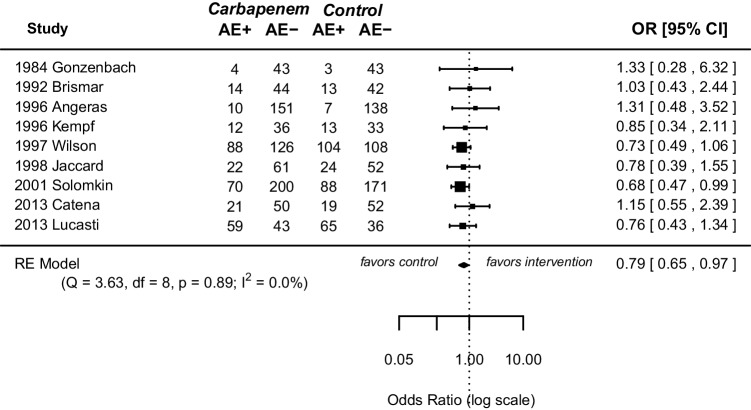
Forest plot of pooled odds ratio with 95% CI for CE vs OA regarding adverse events

All included RCTs reported on adverse events. Slightly lower rates were observed in the carbapenem group (OR 0.79, 95% CI [0.65; 0.97], *p* = 0.022) (Fig. 3). Heterogeneity among studies was low (*I*^2^ = 0.00, *p* = 0.89). Detailed information on adverse events is summarized in Supplemental Table 2.

**Fig. 4:**
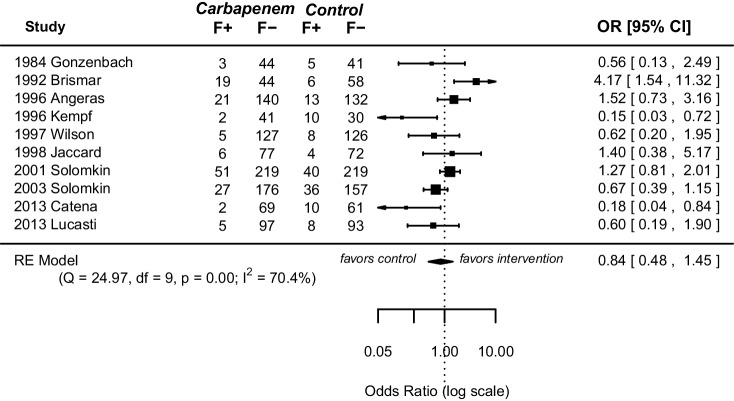
Forest plot of pooled odds ratio with 95% CI for CE vs OA regarding treatment failure

**Fig. 5:**
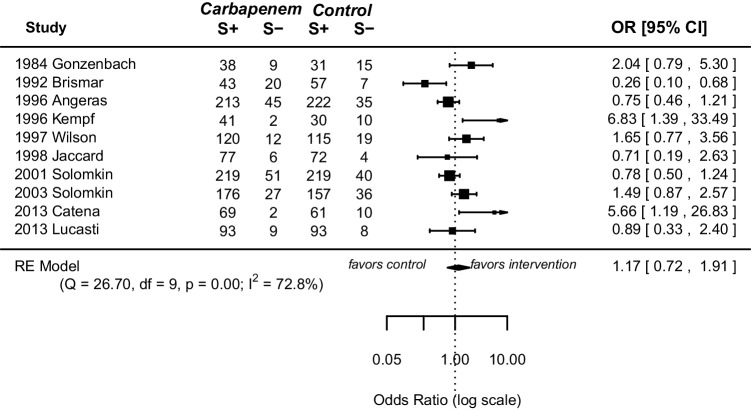
Forest plot of pooled odds ratio with 95% CI for CE vs OA regarding treatment success

All included RCTs reported on treatment failure and treatment success respectively. Meta-analysis of treatment failure and success revealed no statically significant differences between both groups (failure: OR 0.84, 95% CI [0.48; 1.45], *p* = 0.52, success: OR 1.17, 95% CI [0.72; 1.91], *p* = 0.53) (Figs. 4 and 5). Study heterogeneity was substantial for both endpoints (failure: *I*^2^ = 70.4%, *p* = 0.00, success: *I*^2^ = 72.8%, *p* = 0.00).

### Quality Assessment

All trials were RCTs of parallel-group, prospective design. Selection bias (random sequence generation and allocation concealment) was low for seven RCTs. Three studies had a high or unclear risk for selection bias (random sequence generation and allocation concealment). Performance bias (blinding of participants and personnel) was considered high in two studies in which selection bias was already considered high. There was a low risk of detection bias among all RCTs. Incomplete outcome data was reported in five studies (attrition bias) and reporting bias was considered low only for one of the ten studies. Detailed information on the multi-level risk of bias assessment using the Cochrane Collaboration risk-of-bias tool for RCTs is given in Supplemental Table 1. Publication bias was considered high as demonstrated by the asymmetrical funnel plots in Supplemental Fig. 1.

## Discussion

In this systematic review and meta-analysis, we report the impact of carbapenem AB therapy on mortality, treatment success, treatment failure, and adverse events in secondary peritonitis/complicated intra-abdominal infections. A total of 10 RCTs were included. Perforated/complicated appendicitis accounted for around 50% of intra-abdominal infections. No differences between CE and OA groups were observed for mortality and treatment success/failure. There were fewer adverse events in the carbapenem group compared to the control group.

Our results are comparable with past evidence. Despite the high incidence and mortality, no clear surgical medical strategy for very severe cases is defined.^15–17^ Several guidelines give recommendations on how to treat these infections.^18,19^ In a meta-analysis from 1997 involving 10 clinical trials, no statistically significant difference in clinical response between carbapenem monotherapy and combinations of antibiotic therapy in intra-abdominal infections was observed.^20^ Recent meta-analyses also found no clear advantage on use of carbapenem antibiotics over tigecyclines and β-Lactam monotherapy.^21,22^ To our knowledge, the present study is the first meta-analysis comparing carbapenem therapy to any other antibiotic treatment for complicated intra-abdominal infections, with 4 pre-specified, clinically relevant endpoints.

One interesting aspect of our meta-analysis is the lower rate of adverse events in carbapenem antibiotics, compared to control drugs. The safety of carbapenems is well established. Development of a rash and nausea are among the most common adverse events, and treatment is discontinued in only around 1.5% due to side effects.^23^ Allergic reactions are rare. The control antibiotics in our meta-analysis include piperacillin/tazobactam and others that likely cause more adverse events.

This meta-analysis has some limitations. The main drawback is that it is based on RCTs with relatively heterogenic outcome definitions, different (control) antibiotics, and study arms. Control antibiotics groups were cefuroxime and metronidazole, piperacillin-tazobactam, ceftazidime/avibactam plus metronidazole, ampicillin-sulbactam, netilmicin plus clindamycin, cefotaxime plus metronidazole, clindamycin/tobramycin, and clinafloxacin. The carbapenem group included meropenem, imipenem/cilastatin, and ertapenem. All studies had a study arm of carbapenem antibiotics and one arm of other antibiotics. Treatment failure/success and adverse events had also relatively heterogenic definitions across the studies. Detailed information on the definitions of treatment success and treatment failure for each RCT are summarized in Supplemental Table 2.

Mortality was assessed without pre-specified cutoffs (e.g., 30-day or 90-day mortality) in all studies, representing another source of heterogeneity. The numbers of available RCTs and patients were relatively small, increasing the possibility for a type 2 error. The long inclusion period represents another source of potential bias, due to shifting treatment paradigms over time. AB treatment has potential long-term effects going beyond short-term treatment outcomes for individual patients, namely development of resistant microorganisms, representing a major burden for patients and health care providers. The impact of AB resistance development was not investigated in the underlying RCTs and hence not meta-analyzed.

Acquired resistance against carbapenems is most often found in *Klebsiella pneumoniae*, with the highest prevalence of up to 12% in long-term care facilities.^24^ Multi-resistant gram-negative bacteria with carbapenem-resistance are commonly only responding to “last-resort” antibiotics such as colistin and tigecycline. Infections with these pathogens are associated with higher mortality compared to their non-resistant counterparts. *Acinetobacter baumanii* and *Pseudomonas aeruginosa* are among the most common species expressing this problematic, multi-drug resistant phenotype.^25,26^

The strength of this meta-analysis is that all available RCTs providing comparative information on the outcome of patients undergoing carbapenem antibiotics versus other antibiotics for complicated intra-abdominal infections were included. PRISMA guidelines were followed carefully, and individual patient level data was used for analysis, to ensure transparency and comparability across studies.

Therefore, the data should be carefully analyzed, interpreted, and applied. The findings of this work may provide useful information for the design of new RCTs and provide evidence for clinical guidelines.

## Conclusion

In this meta-analysis, all relevant RCTs studies providing comparative information on the outcome of patients undergoing CE antibiotic therapy in CII/SP were included. There is no strong evidence to support CE AB therapy over other AB regimen in this context.

## Supplementary Information

Below is the link to the electronic supplementary material.

Supplementary file1 (DOCX 16 KB)

Supplementary file2 (DOCX 16 KB)

Supplementary file3 (DOCX 17 KB)

Supplementary file4 (PDF 270 KB)

## References

[1] Blot S, Antonelli M, Arvaniti K (2019). Epidemiology of intra-abdominal infection and sepsis in critically ill patients: “AbSeS”, a multinational observational cohort study and ESICM Trials Group Project. Intensive Care Med..

[2] Page MJ, McKenzie JE, Bossuyt PM, Boutron I, Hoffmann TC, Mulrow CD, et al. The PRISMA 2020 statement: an updated guideline for reporting systematic reviews. BMJ. 2021;372:n71. 10.1136/bmj.n71 10.1136/bmj.n71 PMC8005924 33782057

[3] Max Heckler, Laura Schlicht, Christoph W. Michalski. Antibiotic treatment after surgery for complicated intraabdominal infections: a systematic review and meta-analysis. PROSPERO 2018 CRD42018108854 Available from: https://www.crd.york.ac.uk/prospero/display_record.php?ID=CRD42018108854 10.1007/s11605-023-05651-7 PMC10267009 36949237

[4] Gonzenbach, H. R., Simmen, H. P., & Amgwerd, R. (1987). Imipenem (N-F-thienamycin) versus netilmicin plus clindamycin. A controlled and randomized comparison in intra-abdominal infections. Annals of Surgery, 205(3), 271–275. 10.1097/00000658-198703000-00009 10.1097/00000658-198703000-00009 PMC1492722 3548611

[5] Brismar, B., Malmborg, A. S., Tunevall, G., Wretlind, B., Bergman, L., Mentzing, L., … Nordlt, C. E. (1992). Treatment of intra-abdominal infections, 36(12), 2766–2773. 10.1128/aac.36.12.2766 PMC245542 1336347

[6] Kanellakopoulou K, Giamarellou H, Papadothomakos P, Tsipras H, Chloroyiannis J, Theakou R, Sfikakis P (1993). Meropenem versus imipenem/cilastatin in the treatment of intraabdominal infections requiring surgery. European Journal of Clinical Microbiology & Infectious Diseases.

[7] Angerås, M. H., Darle, N., Hamnström, K., Ekelund, M., Engström, L., Takala, J., … Holme, J. B. (1996). A comparison of imipenem/cilastatin with the combination of cefuroxime and metronidazole in the treatment of intra-abdominal infections. Scandinavian Journal of Infectious Diseases, 28(5), 513–518. 10.3109/00365549609037950 10.3109/00365549609037950 8953684

[8] Kempf P, Bauernfeind A, Müller A, Blum J (1996). Meropenem monotherapy versus cefotaxime plus metronidazole combination treatment for serious intra-abdominal infections. Infection.

[9] Wilson SE (1997). Results of a randomized, multicenter trial of meropenem versus clindamycin/tobramycin for the treatment of intra-abdominal infections. Clinical Infectious Diseases.

[10] Jaccard, C., Troillet, N., Harbarth, S., Zanetti, G., Aymon, D., Schneider, R., … Comett, A. (1999). Erratum: prospective randomized comparison of imipenem-cilastatin and piperacillin-tazobactam in nosocomial pneumonia or peritonitis (Antimicrobial Agents and Chemotherapy (1998) 42:11 (2666–2972)). Antimicrobial Agents and Chemotherapy, 43(3), 726. 10.1128/AAC.43.3.726 10.1128/aac.42.11.2966 PMC105974 9797234

[11] Solomkin, J. S., Wilson, S. E., Christou, N. V., Rotstein, O. D., Dellinger, E. P., Bennion, R. S., … Tack, K. (2001). Results of a clinical trial of clinafloxacin versus imipenem/cilastatin for intraabdominal infections. Annals of Surgery, 233(1), 79–87. 10.1097/00000658-200101000-00013 10.1097/00000658-200101000-00013 PMC1421170 11141229

[12] Solomkin, J. S., Yellin, A. E., Rotstein, O. D., Christou, N. V., Dellinger, E. P., Tellado, J. M., … Teppler, H. (2003). Ertapenem versus piperacillin/tazobactam in the treatment of complicated intraabdominal infections. Annals of Surgery, 237(2), 235–245. 10.1097/01.sla.0000048551.32606.73 10.1097/01.SLA.0000048551.32606.73 PMC1522141 12560782

[13] Catena, F., Vallicelli, C., Ansaloni, L., Sartelli, M., Di Saverio, S., Schiavina, R., … Pinna, A. D. (2013). T.E.A. Study: three-day ertapenem versus three-day Ampicillin-Sulbactam. BMC Gastroenterology, 13(1). 10.1186/1471-230X-13-76 10.1186/1471-230X-13-76 PMC3660242 23631512

[14] Lucasti C, Popescu I, Ramesh MK, Lipka J, Sable C (2013). Comparative study of the efficacy and safety of ceftazidime/avibactam plus metronidazole versus meropenem in the treatment of complicated intra-abdominal infections in hospitalized adults: results of a randomized, double-blind, phase II trial. Journal of Antimicrobial Chemotherapy.

[15] Ross JT, Matthay MA, Harris HW. Secondary peritonitis: principles of diagnosis and intervention. BMJ. 2018;361:k1407.10.1136/bmj.k1407 10.1136/bmj.k1407 PMC6889898 29914871

[16] Ordoñez CA, Puyana JC (2006). Management of peritonitis in the critically ill patient. Surg Clin North Am..

[17] Sartelli M, Weber DG, Ruppé E, Bassetti M, Wright BJ, Ansaloni L, Catena F, Coccolini F, Abu-Zidan FM, Coimbra R, Moore EE, Moore FA, Maier RV, De Waele JJ, Kirkpatrick AW, Griffiths EA, Eckmann C, Brink AJ, Mazuski JE, May AK, Sawyer RG, Mertz D, Montravers P, Kumar A, Roberts JA, Vincent JL, Watkins RR, Lowman W, Spellberg B, Abbott IJ, Adesunkanmi AK, Al-Dahir S, Al-Hasan MN, Agresta F, Althani AA, Ansari S, Ansumana R, Augustin G, Bala M, Balogh ZJ, Baraket O, Bhangu A, Beltrán MA, Bernhard M, Biffl WL, Boermeester MA, Brecher SM, Cherry-Bukowiec JR, Buyne OR, Cainzos MA, Cairns KA, Camacho-Ortiz A, Chandy SJ, Che Jusoh A, Chichom-Mefire A, Colijn C, Corcione F, Cui Y, Curcio D, Delibegovic S, Demetrashvili Z, De Simone B, Dhingra S, Diaz JJ, Di Carlo I, Dillip A, Di Saverio S, Doyle MP, Dorj G, Dogjani A, Dupont H, Eachempati SR, Enani MA, Egiev VN, Elmangory MM, Ferrada P, Fitchett JR, Fraga GP, Guessennd N, Giamarellou H, Ghnnam W, Gkiokas G, Goldberg SR, Gomes CA, Gomi H, Guzmán-Blanco M, Haque M, Hansen S, Hecker A, Heizmann WR, Herzog T, Hodonou AM, Hong SK, Kafka-Ritsch R, Kaplan LJ, Kapoor G, Karamarkovic A, Kees MG, Kenig J, Kiguba R, Kim PK, Kluger Y, Khokha V, Koike K, Kok KY, Kong V, Knox MC, Inaba K, Isik A, Iskandar K, Ivatury RR, Labbate M, Labricciosa FM, Laterre PF, Latifi R, Lee JG, Lee YR, Leone M, Leppaniemi A, Li Y, Liang SY, Loho T, Maegele M, Malama S, Marei HE, Martin-Loeches I, Marwah S, Massele A, McFarlane M, Melo RB, Negoi I, Nicolau DP, Nord CE, Ofori-Asenso R, Omari AH, Ordonez CA, Ouadii M, Pereira Júnior GA, Piazza D, Pupelis G, Rawson TM, Rems M, Rizoli S, Rocha C, Sakakushev B, Sanchez-Garcia M, Sato N, Segovia Lohse HA, Sganga G, Siribumrungwong B, Shelat VG, Soreide K, Soto R, Talving P, Tilsed JV, Timsit JF, Trueba G, Trung NT, Ulrych J, van Goor H, Vereczkei A, Vohra RS, Wani I, Uhl W, Xiao Y, Yuan KC, Zachariah SK, Zahar JR, Zakrison TL, Corcione A, Melotti RM, Viscoli C, Viale P. Antimicrobials: a global alliance for optimizing their rational use in intra-abdominal infections (AGORA). World J Emerg Surg. 2016 Jul 15;11:33. 10.1186/s13017-016-0089-y. Erratum in: World J Emerg Surg. 2017 Aug 2;12 :35. PMID: 27429642; PMCID: PMC4946132. 10.1186/s13017-016-0089-y PMC4946132 27429642

[18] Eckmann C, Dryden M, Montravers P (2011). Antimicrobial treatment of “complicated” intra-abdominal infections and the new IDSA guidelines? A commentary and an alternative European approach according to clinical definitions. Eur J Med Res.

[19] Solomkin JS, Mazuski JE, Bradley JS (2010). Diagnosis and management of complicated intra-abdominal infections in adults and children: guidelines by the Surgical Infection Society and the Infectious Diseases Society of America. Clin Inf Dis..

[20] Nguyen CP, Dan Do TN, Bruggemann R, Ten Oever J, Kolwijck E, Adang EMM, Wertheim HFL. Clinical cure rate and cost-effectiveness of carbapenem-sparing beta-lactams vs. meropenem for Gram-negative infections: a systematic review, meta-analysis, and cost-effectiveness analysis. Int J Antimicrob Agents. 2019 Dec;54(6):790–797. 10.1016/j.ijantimicag.2019.07.003. Epub 2019 Jul 5. PMID: 31284041. 10.1016/j.ijantimicag.2019.07.003 31284041

[21] Chen L, Liang X, Jiang J, Li X, Li Y. Carbapenems vs tigecycline for the treatment of complicated intra-abdominal infections: a Bayesian network meta-analysis of randomized clinical trials. *Medicine (Baltimore)*. 2019;98(40):e17436. 10.1097/MD.0000000000017436 10.1097/MD.0000000000017436 PMC6783191 31577763

[22] Li Y, Chen L, Jiang J, Li X, Huang T, Liang X. Carbapenems vs β-lactam monotherapy or combination therapy for the treatment of complicated intra-abdominal infections: systematic review and meta-analysis of randomized controlled trials. *Open Forum Infect Dis*. 2019;6(10):ofz394. Published 2019 Sep 9. 10.1093/ofid/ofz394 10.1093/ofid/ofz394 PMC6786516 31660356

[23] Zhanel GG, Wiebe R, Dilay L (2007). Comparative review of the carbapenems. Drugs.

[24] Kuehn BM (2013). “Nightmare” bacteria on the rise in US hospitals, long-term care facilities. JAMA.

[25] Kaye, K. S., Marchaim, D., Thamlikitkul, V., Carmeli, Y., Chiu, C.-H., Daikos, G., Dhar, S., Durante-Mangoni, E., Gikas, A., Kotanidou, A., Paul, M., Roilides, E., Rybak, M., Samarkos, M., Sims, M., Tancheva, D., Tsiodras, S., Kett, D., Patel, G., … Pogue, J. M. (2023). Colistin monotherapy versus combination therapy for carbapenem-resistant organisms. *NEJM Evidence*, *2*(1). 10.1056/evidoa2200131 10.1056/evidoa2200131 PMC10398788 37538951

[26] Codjoe FS, Donkor ES (2018). Carbapenem resistance: a review. Med. Sci..

