# Supplementary material for: Carbapenem Antibiotics Versus Other Antibiotics for Complicated Intra-abdominal Infections: a Systematic Review and Patient-Level Meta-analysis of Randomized Controlled Trials (PROSPERO CRD42018108854)
Source: J Gastrointest Surg. 2023 Mar 22;27(6):1208–15. doi: 10.1007/s11605-023-05651-7 (PMC10267009; doi:10.1007/s11605-023-05651-7)
Supplement: Supplementary file 1 — Supplementary file1 (DOCX 16 KB) [file 11605_2023_5651_MOESM1_ESM.docx]

| **Author** | **Year** | **Duration of FU** | **Success Definition** | **Failure Definition** |
| --- | --- | --- | --- | --- |
| *Gonzenbach* | *1984* | *n.s.* | *no infection within 3 weeks* | *reoperation due to purulent collection; death* |
| *Brismar* | *1992* | *4-6 weeks after end of treatment* | *asymptomatic with no evidence of infection at the time of posttherapy evaluation* | *no demonstrable response to therapy* |
| *Kanellakopoulou* | *1993* | *30 days after end of treatment* | *n.s.* | *n.s.* |
| *Angeras* | *1996* | *30 days after hospital stay* | *global efficacy very good or good (based on clinical signs of infection)* | *global efficacy poor or very poor* |
| *Kempf* | *1996* | *2-4 weeks after end of treatment* | *complete remission of local and systemic signs and symptoms of infection without the addition of other antibiotics and without subjective or objective evidence of recurrence* | *no improvement or with a deterioration of signs and symptoms* |
| *Wilson* | *1997* | *4-6 weeks after end of treatment* | *Pretreatment pathogens eradicated or clinical response satisfactory* | *no eradication of pretreatment pathogens or worsened signs of infection under treatment* |
| *Jaccard* | *1998* | *2-4 weeks after end of treatment* | *n.s.* | *any further antibiotic treatment or surgery for peritonitis within 7 days after the end of treatment* |
| *Solomkin* | *2001* | *n.s.* | *no evidence of infection by clinical, laboratory, and radiographic criteria* | *defined as persisting or recurrent infection in the abdomen, documented by the findings at percutaneous or surgical reintervention, or postsurgical wound infection* |
| *Solomkin* | *2003* | *4-6 weeks after end of therapy* | *n.s.* | *persisting or recurrent infection within the abdomen, postsurgical wound infection; death related to ongoing intraabdominal infection, treatment with additional antibiotics for undocumented intraabdominal infection during the study period* |
| Catena | 2013 | *n.s.* | *no signs or symptoms of infection and no further need for antimicrobial therapy* | *no improvement, progression of infection, or death due to infection or indicating recurrence between cessation of antibiotic therapy and follow-up* |
| Lucasti | 2013 | *4-6 weeks after end of therapy* | *complete resolution or significant improvement of signs/symptoms of infection with no requirement for additional antibiotics or surgery* | *n.s.* |
