# Supplementary material for: Carbapenem Antibiotics Versus Other Antibiotics for Complicated Intra-abdominal Infections: a Systematic Review and Patient-Level Meta-analysis of Randomized Controlled Trials (PROSPERO CRD42018108854)
Source: J Gastrointest Surg. 2023 Mar 22;27(6):1208–15. doi: 10.1007/s11605-023-05651-7 (PMC10267009; doi:10.1007/s11605-023-05651-7)
Supplement: Supplementary file 2 — Supplementary file2 (DOCX 16 KB) [file 11605_2023_5651_MOESM2_ESM.docx]

| **Author** | **Year** | **Mortality** | | **Adverse Events** | |
| --- | --- | --- | --- | --- | --- |
| *Gonzenbach* | *1984* | *not reported* | | ***A****: C. diff. infection, eosinophilia , candida in respiratory tract* | ***B****: elevation of serum creatinine, eosinophilia* |
| *Brismar* | *1992* | *n.s.* | | ***A****: diarrhea, nausea* | ***B****: diarrhea, nausea* |
| *Kanellakopoulou* | *1993* | *n.s.* | | ***A****: elevated liver transaminase level, thrombocythemia* | ***B****: elevated liver transaminase level, thrombocythemia* |
| *Angeras* | *1996* | *n.s.* | | ***A****: fever, pruritus, rash, laboratory adverse event* | ***B****: rash, laboratory adverse event* |
| *Kempf* | *1996* | ***A****:cardiogenic shock, cardiac failure, myocardial infarction* | ***B****:PE, ARDS, septic shock* | ***A****: headache, diarrhea, mild changes shown by liver function tests, moderately increased creatinine* | ***B****: increased liver transaminase level* |
| *Wilson* | *1997* | ***A****: respiratory failure* | ***B****: cardiac tamponade* | *increased liver enzymes, rash, thrombocythemia, diarrhea* | |
| *Jaccard* | *1998* | *n.s.* | | *diarrhea, allergic reaction, C. albicans infection, C. diff. colitis, nephrotoxicity, hepatotoxicity, hematotoxicity, colonization by a resistant organism* | |
| *Solomkin* | *2001* | *uncontrolled infection and multisystem organ failure, malignancy, AMI, PE* | | ***A****: diarrhea, nausea, rash, abnormal results on liver function tests* | ***B****: diarrhea, hypoglycemia, vaginal moniliasis, nausea* |
| *Solomkin* | *2003* | *n.s.* | | ***A****: C. diff. colitis, rash, confusion, thrombocytopenia, grand mal seizure* | ***B****: rash, deep vein thrombosis, infused vein complication, confusion , jaundice, hypertension, seizure disorder* |
| *Catena* | *2013* | ***A****: AMI* | ***B****: PE* | *n.s.* | |
| *Lucasti* | *2013* | ***A****: peritonitis, pneumonia, thrombocytopenia* | ***B****: multiorgan failure, sepsis, cardiac arrest* | *nausea, vomiting, abdominal pain, pyrexia, wound secretion, cough, laboratory adverse events* | |
