# Supplementary material for: Carbapenem Antibiotics Versus Other Antibiotics for Complicated Intra-abdominal Infections: a Systematic Review and Patient-Level Meta-analysis of Randomized Controlled Trials (PROSPERO CRD42018108854)
Source: J Gastrointest Surg. 2023 Mar 22;27(6):1208–15. doi: 10.1007/s11605-023-05651-7 (PMC10267009; doi:10.1007/s11605-023-05651-7)
Supplement: Supplementary file 3 — Supplementary file3 (DOCX 17 KB) [file 11605_2023_5651_MOESM3_ESM.docx]

| ***Included studies*** | ***Selection bias (Random sequence generation)*** | ***Selection bias (Allocation concealment)*** | ***Performance bias***  ***(Blinding of participants and personnel)*** | ***Detection bias***  ***(Blinding of outcome assessment)*** | ***Attrition Bias (incomplete outcome data)*** | ***Reporting Bias (Selective reporting)*** | ***Other bias*** |
| --- | --- | --- | --- | --- | --- | --- | --- |
| 1984 Gonzenbach | **+** | **?** | **?** | **?** | **+** | **?** | **?** |
| 1995 Brismar | **+** | **+** | **+** | **+** | **+** | **?** | **+** |
| 1996 Angeras | **-** | **?** | **-** | **+** | **-** | **-** | **-** |
| 1996 Kempf | **-** | **-** | **-** | **+** | **+** | **?** | **?** |
| 1997 Wilson | **+** | **+** | **+** | **+** | **+** | **?** | **+** |
| 1998 Jaccard | **+** | **+** | **+** | **+** | **+** | **?** | **-** |
| 2001 Solomkin | **?** | **+** | **+** | **+** | **?** | **?** | **?** |
| 2003 Solomkin | **-** | **+** | **+** | **+** | **-** | **?** | **-** |
| 2013 Catena | **+** | **+** | **+** | **+** | **?** | **+** | **?** |
| 2013 Lucasti | **+** | **+** | **+** | **+** | **?** | **?** | **-** |
