# Supplementary figures and images for: Carbapenem Antibiotics Versus Other Antibiotics for Complicated Intra-abdominal Infections: a Systematic Review and Patient-Level Meta-analysis of Randomized Controlled Trials (PROSPERO CRD42018108854)
Source: J Gastrointest Surg. 2023 Mar 22;27(6):1208–15. doi: 10.1007/s11605-023-05651-7 (PMC10267009; doi:10.1007/s11605-023-05651-7)

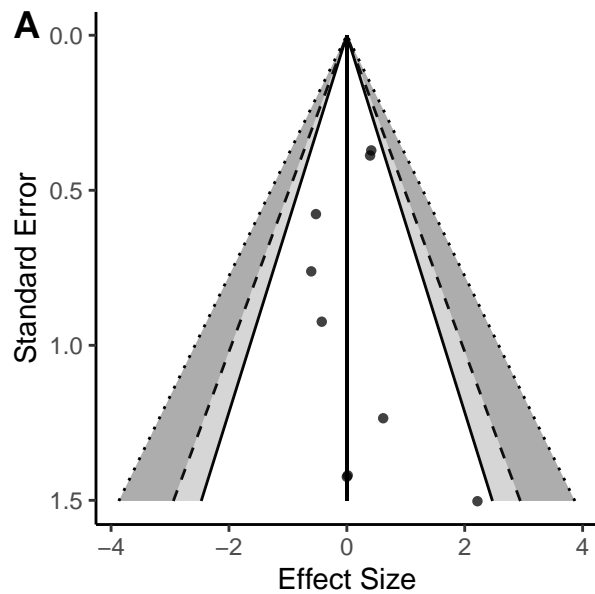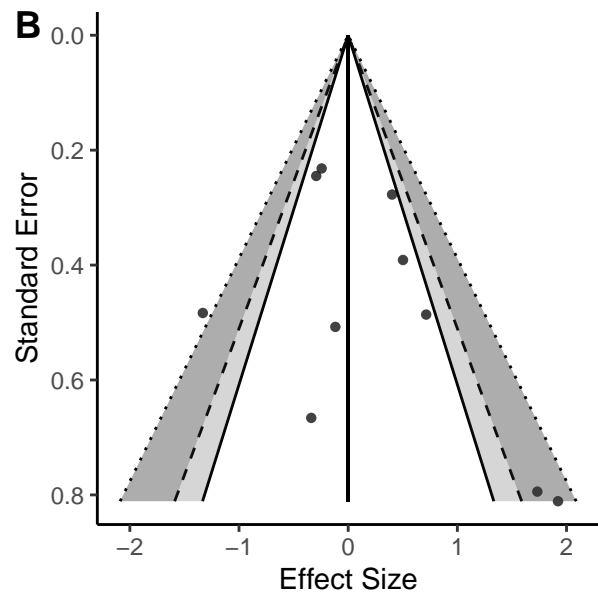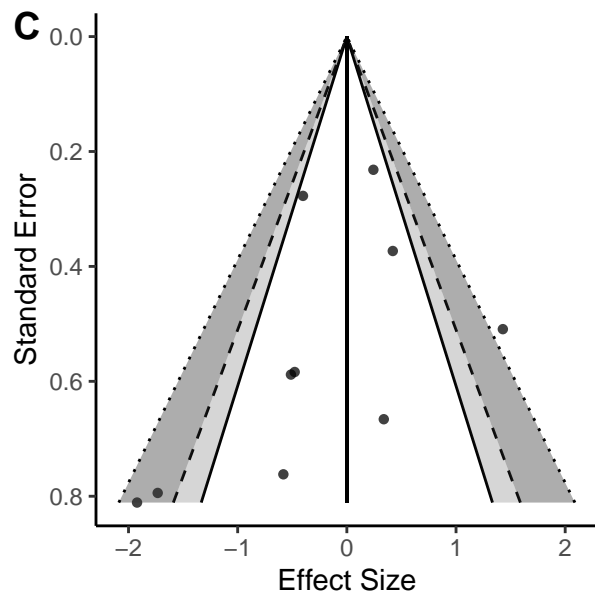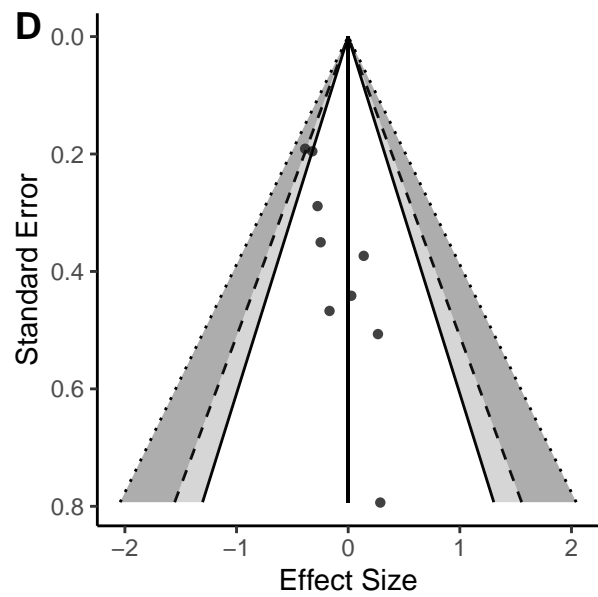

Supplement: Supplementary file 4 — Supplementary file4 (PDF 270 KB) [file 11605_2023_5651_MOESM4_ESM.pdf]
